# Combined effect of branched-chain amino acids and taurine supplementation on delayed onset muscle soreness and muscle damage in high-intensity eccentric exercise

**DOI:** 10.1186/1550-2783-10-51

**Published:** 2013-11-06

**Authors:** Song-Gyu Ra, Teruo Miyazaki, Keisuke Ishikura, Hisashi Nagayama, Shoichi Komine, Yoshio Nakata, Seiji Maeda, Yasushi Matsuzaki, Hajime Ohmori

**Affiliations:** 1 Graduate School of Comprehensive Human Sciences, University of Tsukuba, Tsukuba, Ibaraki 305-8574, Japan; 2 Research Fellow of the Japan Society for the Promotion of Sciences, Chiyoda-ku, Tokyo 102-0083, Japan; 3 Joint Research Center, Tokyo Medical University Ibaraki Medical Center, Ami, Ibaraki 300-0395, Japan; 4 Sports Research and Development Core, University of Tsukuba, Tsukuba, Ibaraki 305-8574, Japan; 5 School of Health and Physical Education, University of Tsukuba, Tsukuba, Ibaraki 305-8574, Japan; 6 Faculty of Medicine, University of Tsukuba, Tsukuba, Ibaraki 305-8574, Japan; 7 Faculty of Health and Sport Sciences, University of Tsukuba, Tsukuba, Ibaraki 305-8574, Japan; 8 Division of Gastroenterology and Hepatology, Department of Internal Medicine, Tokyo Medical University Ibaraki Medical Center, Ami, Ibaraki 300-0395, Japan

**Keywords:** Double-blind study, Amino acids, Combined supplementation, BCAA, Taurine, DOMS

## Abstract

**Background:**

Previous studies have evaluated the effectiveness of branched-chain amino acid (BCAA) supplementation for preventing delayed onset muscle soreness (DOMS) and muscle damage induced by eccentric exercise, their findings have been inconclusive. Since taurine has anti-inflammatory and anti-oxidative effects, the present study investigated the combined effect of BCAA and taurine on DOMS and muscle damage.

**Methods:**

Thirty-six untrained male subjects (22.5 ± 3.8 years) were assigned to four groups (placebo + placebo [placebo], BCAA + placebo, placebo + taurine, and BCAA + taurine [combined]) and given a combination of 3.2 g BCAA (or placebo) and 2.0 g taurine (or placebo), three times a day, for two weeks prior to and three days after eccentric elbow flexor exercises. DOMS and muscle damage in the biceps brachii were subjectively and objectively evaluated using the visual analogue scale (VAS), upper arm circumference (CIR), and blood parameters (creatine kinase, lactate dehydrogenase [LDH], aldolase, and 8-hydroxydeoxyguanosine [8-OHdG]).

**Results:**

In the combined group, VAS and 8-OHdG two days after exercise, CIR two and three days after exercise and LDH from one to three days after exercise were significantly lower than the placebo group. The area under the curve from before exercise to four days later for CIR, LDH, and aldolase was also significantly lower in the combined group than in the placebo group.

**Conclusion:**

A combination of 3.2 g BCAA and 2.0 g taurine, three times a day, for two weeks prior to and three days after exercise may be a useful nutritional strategy for attenuating exercise-induced DOMS and muscle damage.

## Introduction

The importance and benefits of regular exercise in maintaining overall health and preventing aging are well known. However, unaccustomed and sudden exercise results in dull pain in the skeletal muscle within hours or days after exercise, which is referred to as delayed onset muscle soreness (DOMS) [1]. DOMS is one of the symptoms of eccentric-exercise (ECC)-induced muscle damage. Muscle damage is characterized as disruption of the membrane by mechanical stress, infiltration of inflammatory cells to the injured tissue, and increased production of inflammatory cytokines [2]. Pain resulting from DOMS leads to a decrease in exercise performance and muscle strength gains for up to three weeks [3].

Branched-chain amino acids (valine, leucine, and isoleucine; BCAAs) are abundant and catabolized in the skeletal muscle, and they help to inhibit protein breakdown [4] and enhance protein synthesis [5]. BCAAs have been reported in many studies to attenuate DOMS and muscle damage induced by exercise [4,6-11]. Shimomura *et al.* reported that BCAA supplementation prior to squat exercises decreased DOMS within a few days after exercise [7,8]. Furthermore, the beneficial effects of BCAA supplementation on DOMS together with the inhibition of muscle damage was also observed for a training program involving trained long-distance runners [4] and in cycling exercise [9,10]. In contrast, a study by Jackman *et al.* found no attenuating effects of BCAA supplementation on DOMS in the quadriceps muscle with the knee extended or on inflammation during the recovery period following high-intensity knee extension exercise, but DOMS was attenuated when measured with the knee flexed [11]. Thus, the positive effects of BCAA supplementation on DOMS and muscle damage were weak in high-intensity exercise. Previous studies have evaluated the combined effects of various nutrients and BCAA supplements on DOMS and muscle damage. Stock *et al.* examined the combined effect of leucine supplementation and a carbohydrate beverage on DOMS and serum muscle damage markers during the recovery period following squat exercises; however, no significant effects were found before or after exercise [12]. Furthermore, the combination of protein (free-form amino acids including BCAA) and carbohydrate supplements given before and after ECC had no effect on muscle damage, loss of strength, or muscle soreness [13]. Therefore, combining BCAAs with other anti-inflammatory nutrients might be beneficial for alleviating DOMS and muscle damage.

Taurine (2-aminoethanesulfonic acid), which is abundant in skeletal muscle, has been reported to have many physiological and pharmacological actions, including membrane stabilization, anti-oxidation, osmoregulation, modulation of ion flux, and control of Ca^2+^ homeostasis, in addition to playing roles as a neurotransmitter and neuromodulator [14]. In particular, it was reported that taurine has a cytoprotective effect against free radical-mediated skeletal muscle injury induced by downhill running in rats [15,16]. The authors also confirmed that oral taurine administration in rats reduces exercise- and drug-induced oxidative stress [17,18]. Interestingly, a multi-nutrient supplement containing BCAA and taurine as well as some vitamin B and plant extracts improved inflammation and joint pain in middle-age individuals [19]. Therefore, we hypothesized that taurine might enhance the beneficial effect of BCAA on DOMS and muscle damage induced by exercise.

In the present study, we investigated by means of a randomized, placebo-controlled, double-blind trial whether a combination of BCAA and taurine supplements can provide an effective nutritional strategy for attenuating DOMS and muscle damage induced by high-intensity exercise in humans.

## Methods

### Subjects and amino acid treatment protocol

This randomized, placebo-controlled, double-blind trial was conducted with 36 male college volunteers who did not have any musculoskeletal disorders and had not partaken in any regular resistance training prior to the study (Table 1). The study was carried out in accordance with the Declaration of Helsinki and was approved by the Human Subjects Committee of the University of Tsukuba. All subjects provided written informed consent.

**Table 1:** Grouping conditions and characteristics of subjects

	**Supply condition**		**Physiological characteristics before experiment**						
**Group**	**BCAA**	**Taurine**	**Age (years)**	**Height (cm)**	**Body W. (kg)**	**Fat (%)**	**Muscle W. (kg)**	**MVC (Nm)**	**CIR (mm)**
***PLCB***	Placebo-1	Placebo-2	22.2 ± 1.1	170.8 ± 1.9	67.5 ± 3.5	18.3 ± 1.9	51.9 ± 1.8	36.5 ± 3.0	257.4 ± 7.6
***BA***	3.2 g	Placebo-2	22.9 ± 1.1	176.7 ± 3.6	73.5 ± 4.3	20.1 ± 1.7	55.3 ± 2.4	41.9 ± 3.8	267.7 ± 7.9
***TAU***	Placebo-1	2.0 g	22.2 ± 0.8	173.7 ± 2.2	65.5 ± 2.7	17.3 ± 1.3	51.7 ± 1.7	39.1 ± 2.4	251.3 ± 7.4
***COMB***	3.2 g	2.0 g	23.1 ± 1.3	174.5 ± 1.8	61.5 ± 1.6	14.2 ± 1.0	50.0 ± 1.3	35.0 ± 2.6	243.4 ± 6.6

Footnote: Data are expressed as means ± S. E. *Abbreviation*: *PLCB*, double-placebo control group; *BA*, branched-chain amino acids and placebo-2 supplement group; *TAU,* taurine and placebo-1 supplement group; *COMB,* combined (taurine and BCAA) supplement group; *Placebo-1 and 2*, placebo of BCAA and taurine supplementation, respectively (3.2g or 2.0g starch mainly), *Body W*., body weight; *Muscle W*., muscle weight; *MVC*, maximal voluntary strength of isometric contraction; *CIR*, upper arm circumference.

**Figure 1:**
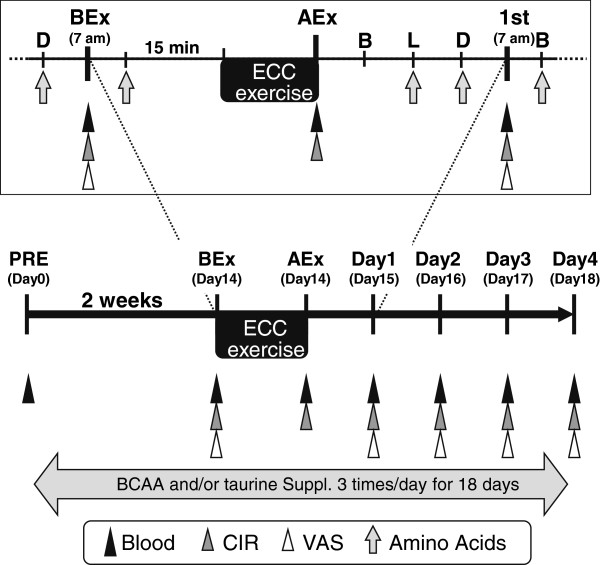
**A schematic illustrating the experimental protocol and time course of the present study.** Participants were supplied with two kinds of sachets consists of combination of BCAA (or placebo of BCAA) and taurine (or placebo of taurine) from 2 weeks before exercise to the end of the experiment. Participants were performed elbow extension as part of ECC in the non-dominant arm using dumbbell weight. Muscle soreness and damage were then monitored for 4 days after ECC. *Abbreviations*: *PRE*, prior to amino acid supplementation; *BEx*, before exercise; *AEx*, immediately after exercise; *Day1-Day4*, 1st to 4th days following exercise; *ECC*, 6 sets of 5 repetitions of eccentric elbow extensions at 90% of maximal isometric strength; *VAS*, visual analogue scale for delayed onset muscle soreness assessment; *CIR*, upper arm circumference; *Blood*, blood sampling; *Amino Acids*, combination of amino acids (BCAA and/or taurine) supplementation; *Suppl.*, supplementation; *B*, breakfast; *L*, lunch; *D*, dinner.

Subjects were randomly and equally divided into the following four groups (*n* = 9 per group): double-placebo control supplementation (PLCB); BCAA and placebo-2 supplementation (BA); taurine and placebo-1 supplementation (TAU); and BCAA and taurine supplementation (COMB). Subjects were orally administered two sachets containing a combination of BCAA (or placebo-1) and taurine (or placebo-2) after every meal for two weeks prior to exercise (Table 1 and Figure 1). We chose this timeframe because previous studies showed a significant increase in muscular taurine concentration following two weeks of taurine administration in rats [18,20], but not after one week in humans [21]. Since the present study was designed as a double-blind trial, the duration of BCAA supplementation prior to exercise was matched to the two-week duration of taurine supplementation. All subjects were instructed to fill out a supplemental checklist after every meal. The BCAA and taurine sachets contained 3.2 g (9.6 g/day) of a BCAA mixture (Ile: Leu: Val = 1:2:1; Aminofeel®, Seikatsu Bunkasya Co. Inc., Chiba, Japan) and 2.0 g (6.0 g/day) of taurine, respectively. The daily doses of BCAA and taurine were based on the doses used in previous studies, which examined the effectiveness of BCAA supplementation on DOMS [9,22] and plasma taurine levels [23]. The daily doses per body weight of BCAA and taurine were 145.7 ± 5.3 (109.5–181.5) and 95.5 ± 2.5 (80.3–116.5) mg/kg (mean ± standard error, range), respectively. The placebo-1 and -2 supplements were compounded to the same volume and color as the BCAA and taurine supplements, respectively, by using similar proportions of starch for the double-blind method (Table 1). Supplementation was continued in a double-blind manner until dinner on the third day after exercise. Evaluation using a visual analogue scale (VAS) and by assessing muscle damage markers was completed on the morning of the fourth day after exercise. No significant differences in physical parameters measured a week before starting supplementation were noted between the groups (Table 1). All subjects were sedentary men who were non-athletes. They were instructed to continue their normal activities and to abstain from any strenuous exercise for at least one month before the experiment. Moreover, they were instructed to continue their usual food intake, not to change the amount or frequency of dietary meat or seafood intake, and not to use any dietary supplements, anti-inflammatory drugs, or anything else that could affect muscle soreness and damage until the end of the study. They were also instructed to abstain from stretching or massage therapy during the experimental period.

### Exercise protocol

Figure 1 outlines the experimental protocol, including the time course corresponding to amino acid supplementation, exercise, and parameter measurement. On the day of exercise, all subjects assembled at our laboratory at 07:00 after fasting overnight. Following blood sampling, they ingested their assigned supplements 15 min prior to performing ECC. After the exercise at 10:00, subjects were supplied with jelly-type food (160 kcal/180 g; Nihon Pharmaceutical Co., Ltd., Toyama, Japan) to minimize protein catabolism due to fasting [24]. The exercise protocol, designed to induce soreness in the elbow flexors, was modified from a previously published method of voluntary ECC [25]. During the week prior to initiating amino acid supplementation, the maximal voluntary strength of isometric contraction (MVC) in the non-dominant arm of each subject was measured at 1.57 rad (90°) of elbow flexion.

For the ECC protocol, subjects were seated on a bench with their arm positioned in front of their body and resting on a padded support, such that their shoulder was secured at a flexion angle of 0.79 rad (45°) and their forearm was maintained in the supinated position throughout the exercise. Subjects were repeatedly weight-loaded upon dumbbell lowering to achieve a 90% MVC (34.3 ± 1.3 Nm). Subjects performed six sets of five repetitions of elbow extension from the flexed position at 90° to the fully extended position slowly over 5 s, while maintaining a constant speed of movement by following a verbal metronome provided by the investigator. After each extension, the investigator returned the dumbbell to the starting position (90°) to prevent excess muscle activation induced by the weight. Subjects were permitted to rest for 3 s between repetitions and for 2 min between sets. The intensity of ECC at 90% MVC was determined on the basis of our preliminary experiments and likely induced natural muscle damage as all subjects found it difficult to lower the dumbbell at a constant speed during the later sets due to decreased muscle function. The subjects also required verbal encouragement from the investigator to maintain constant speed.

### Blood parameters of muscle damage

Blood samples were collected from the antecubital vein at seven different time points: prior to amino acid supplementation, before exercise, immediately after exercise, at one to four days after exercise (Day1–4) (Figure 1). On the day of exercise, blood was collected before supplement intake, and exercise was started thereafter. Immediately after exercise, blood was collected again. In the four days following exercise, blood was collected at 07:00 before breakfast and amino acid intake. Serum was centrifuged for 30 min after the formation of a solid clot, and the plasma was immediately separated. The serum activities of creatine kinase (CK), lactate dehydrogenase (LDH), and aldolase were analyzed and used as parameters of muscle damage, as described in the Japan Society of Clinical Chemistry consensus methods. Serum levels of 8-hydroxydeoxyguanosine (8-OHdG), a marker of oxidative stress-induced DNA damage, were measured before exercise and on Day 2 after exercise by competitive enzyme-linked immunosorbent assay (Highly Sensitive 8-OHdG Check ELISA kit; Japan Institute for the Control of Aging, Fukuroi, Japan) after purification with a 10-kDa filter (Nanosep®; Pall Corporation, NY, US). Additionally, plasma taurine and BCAA concentrations were measured using an automatic amino acid analyzer (JLC-300V; JEOL, Tokyo, Japan) after de-proteination with a two-fold volume of 3% sulphosalicylic acid [26]. Plasma albumin concentrations were analyzed by the bromocresol green method (Albumin II-HA test Wako; Wako Pure Chemicals, Osaka, Japan).

### Subjective assessments of muscle soreness

Subjective assessment of elbow flexor soreness in the biceps brachii muscle was surveyed using the VAS, which consisted of a 100-mm line with “no pain” at one end and “extreme pain” at the other end [25]. VAS scores were measured before exercise and on Days 1–4 with the arm in the extended position. Specifically, the exercised arm was placed on a table in the seated position and the investigator passively extended the elbow joint to test the perception of soreness. Because the degree of soreness in the extended arm position was influenced by the technique of the investigator, the same investigator performed all measurements to avoid inter-investigator measurement error. The test-retest reliability determined using an intraclass correlation coefficient (ICC) was 0.98.

### Indirect marker of muscle damage via physical parameters

The upper arm circumference (CIR) was used as an indirect marker of muscle damage and measured before exercise, after exercise, and on Days 1–4 (Figure 1). CIR was measured at five points 3, 5, 7, 9, and 11 cm proximal to the elbow joint on a relaxed arm in the standing position using a constant-tension tape. To avoid daily variations in the measurement position, these sites on the upper arm were marked with a semi-permanent ink pen during the first testing session. CIR was measured in duplicate and the mean value of each point was used for analysis. The values immediately after exercise and on Days 1–4 were presented as the differences from the values before exercise. The test-retest reliability determined using an ICC for CIR was 0.99.

### Statistical analysis

Data are expressed as means ± SE. The values of CK, LDH, aldolase, VAS, and CIR are presented as raw values and as the area under the curve (AUC) during the experimental period. The AUC was calculated as the sum of four or five trapezoid areas separated by each measurement time point. At each point, the effects of each supplement protocol on the measured outcomes were determined by one-way analysis of variance (ANOVA) followed by Tukey’s test or the non-parametric Wilcoxon *post hoc* test. Significant differences between two points and between multiple points within the same group were analyzed using Student’s paired *t*-test and repeated-measures ANOVA with Dunnett’s *post hoc* multiple comparison test, respectively. Significant differences (two-tailed) were set at *P* < 0.05. Analysis was conducted using SPSS software version 18.0 for Windows (IBM, Chicago, IL).

## Results

### Plasma amino acid concentrations

**Figure 2:**
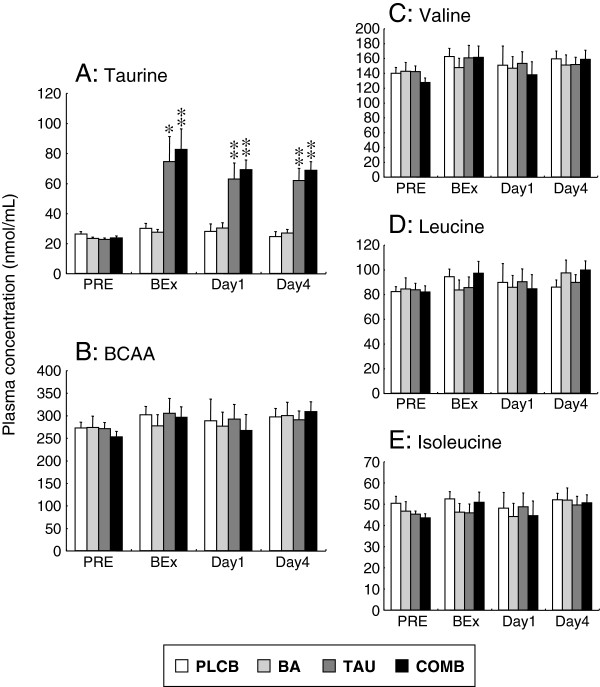
**Plasma taurine (A), BCAA (B), valine (C), leucine (D) and isoleucine (E) concentrations.***Abbreviations*: *PLCB*, placebo supplementation group; *BA*, BCAA supplementation group; *TAU*, taurine supplementation group; *COMB*, combined (BCAA + taurine) supplementation group; *PRE*, prior to amino acid supplementation; *BEx*, before exercise; *Day1*, 1st day after exercise; *Day4*, 4th day after exercise. Data are expressed as means ± S.E. *P < 0.05, **P < 0.01 versus the PLCB and BA groups by one-way ANOVA.

Figure 2 shows the plasma concentrations of taurine, total BCAA, and individual BCAAs prior to amino acid supplementation, before exercise, and on Days 1 and 4. Prior to supplementation, there were no significant differences in plasma taurine concentration among the groups (Figure 2A). Before exercise and on Days 1 and 4, the plasma taurine concentration in the TAU and COMB groups was significantly increased compared with that in the PLCB and BA groups (Figure 2A). No significant differences in the plasma concentrations of total BCAA and individual BCAAs (valine, leucine, or isoleucine) were observed among the groups at any time points (Figure 2B-E).

The plasma albumin concentration (4.0–5.3 g/dL) in all subjects was within the normal range, and no significant differences were observed between the groups throughout the experimental period (data not shown).

### Delayed onset muscle soreness following eccentric exercise

**Figure 3:**
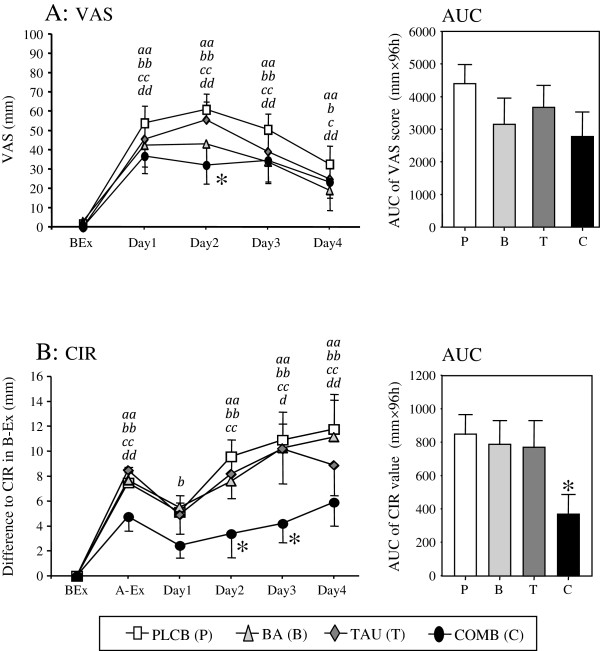
**VAS score (A) and CIR (B) throughout the experiment.** VAS score was used as subjectively assessment of muscle soreness in the exercised arm. CIR value as an indirect marker of muscle damage is presented as differences from the respective BEx. Both parameters were also shown as the AUC from BEx to Day4. *Abbreviations*: *VAS*, visual analog scale; *AUC*, area under the curve; *CIR*, upper arm circumference. Data are expressed as means ± S.E. *P < 0.05 versus the PLCB group by one-way ANOVA. ^*a,b,c,d*^ show the significant difference compared with the corresponding Pre in the PLCB, BCAA, TAU, and COMB groups, respectively, and single and double characters mean *P* < 0.05 and *P* < 0.01, respectively, by repeated measures ANOVA.

Figure 3A shows the VAS scores for subjective DOMS assessment. The VAS scores in all groups were significantly higher on Day 1 compared with before exercise. The VAS scores in the BA and COMB groups peaked on Day 1 while those in the PLCB and TAU groups peaked on Day 2. The increased VAS scores in all groups declined by Day 4. In the COMB group, the VAS scores on Day 2 were significantly lower than in the PLCB group.

### Indirect marker of muscle damage

CIR as an indirect marker of muscle damage is shown in Figure 3B. CIR differences increased significantly and immediately after exercise in all groups and declined by Day 1. Thereafter, the CIR differences in all groups increased significantly until the end of the experimental period. In the COMB group, the CIR differences were lower than in the other groups throughout the experimental period, with significant differences on Days 2 and 3 compared with the PLCB group. Additionally, the AUC of the CIR differences in the COMB group was significantly lower than in the PLCB group. No significant differences in CIR were observed among the PLCB, BA, or TAU groups throughout the experimental period.

### Blood parameters of muscle damage

**Figure 4:**
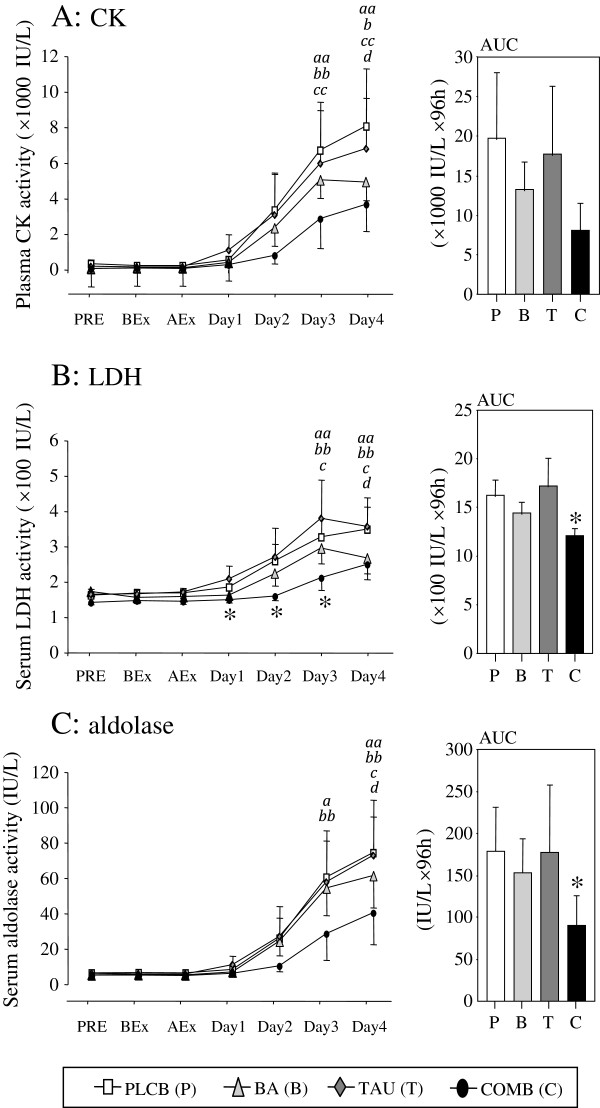
**Serum activities of CK (A), LDH (B), and aldolase (C) throughout the experimental period.** The AUC of these parameters calculated through the experimental period was also shown. *Abbreviations*: *CK*, creatine kinase; *LDH*, lactate dehydrogenase; *PLCB [****P****]*, placebo supplementation group; *BA [****B****]*, BCAA supplementation group; *TAU [****T****]*, taurine supplementation group; *COMB [****C****]*, combined (BCAA + taurine) supplementation group. Data are expressed as means ± S.E. *P < 0.05 versus the PLCB group by one-way ANOVA. ^*a,b,c,d*^ show the significant difference compared with the corresponding Pre in the PLCB, BCAA, TAU, and COMB groups, respectively, and single and double characters mean *P* < 0.05 and *P* < 0.01, respectively, analyzed by repeated measures ANOVA.

The serum enzyme activities throughout the experimental period of CK, LDH, and aldolase, which serve as blood parameters of muscle damage, are presented in Figure 4. All serum markers remained unchanged in all groups until Day 1 and then increased from Day 2 to Day 4.

Serum CK activity in the PLCB, BA, and TAU groups was significantly higher on Days 3 and 4 compared with before exercise (Figure 4A). In the COMB group, a significant difference in CK activity compared with before exercise was found only on Day 4. Statistically significant differences among all groups was not found at any points throughout the experiment due to the large variance between individuals. Serum LDH activity from Day 1 to Day 3 and the AUC were significantly lower in the COMB group than in the PLCB group (Figure 4B). Similarly, serum aldolase activity in the COMB group was lower than in other groups, and a significant difference was noted only before exercise on Day 4 (Figure 4C). The AUC of aldolase was significantly lower in the COMB group than in the PLCB group.

**Figure 5:**
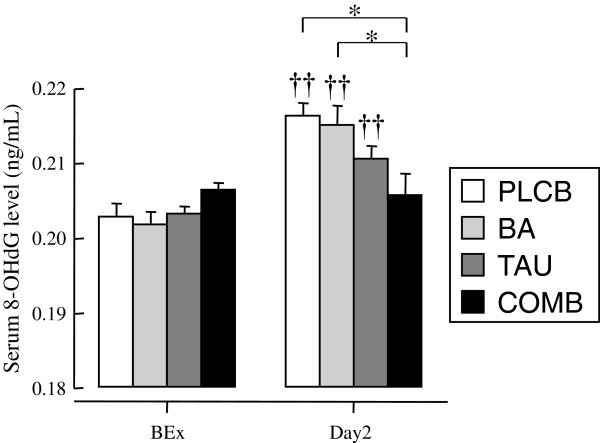
**Serum 8-OHdG level at BEx and Day2.***Abbreviations*: *8-OHdG*, 8-hydroxydeoxyguanosine; *BEx*, before exercise; *Day2*, 2nd day after exercise. Data are shown as means ± S.E. *P < 0.05 above the column with bar shows the significant difference analyzed by one-way ANOVA. ††*P* < 0.01 on the column without bar shows the significant difference compared to the respective values in the BEx by paired Student’s *t*-test.

Figure 5 shows serum 8-OHdG levels before exercise and on Day 2. Before exercise, there was no significant difference in serum 8-OHdG levels between any groups. Serum 8-OHdG levels in the PLCB, BA, and TAU groups were significantly increased on Day 2 compared with before exercise. On Day 2, 8-OHdG levels were significant lower in the COMB group than in the PLCB and BA groups.

## Discussion

Numerous studies have confirmed the effectiveness of BCAA supplementation on DOMS and muscle damage [4,7-11]. However, the attenuating effects of BCAA on the DOMS and muscle damage were occasionally limited, especially in case of intensive exercise. Consequently, more effective nutritional strategies need to be discovered. In the present study, the effects of BCAA supplementation combined with taurine on a highly intense ECC-induced DOMS and muscle damage were investigated via a randomized, placebo-controlled, and double-blind trial, because taurine was reported to decrease oxidative stress induced by ECC [16]. In ECC-induced DOMS and muscle damage, subjective and objective parameters including VAS scores, CIR, and serum levels of LDH and 8-OHdG were significantly improved by the combination of BCAA and taurine supplementation. This combined supplementation also tended to improve serum CK and aldolase activities, but not significantly. These parameters, especially serum CK activity, have a high degree of individual biological variability, and it is difficult to demonstrate a statistically significant difference between the small number of subjects [3]. Overall, the present study demonstrated that combined supplementation with BCAA and taurine is beneficial for reducing ECC-induced DOMS and muscle damage. However, it was impossible to determine whether the combined effects were due to the synergistic effect of both BCAA and taurine or the sum of the individual effects.

Compared with the effectiveness of BCAA supplementation on exercise-induced muscle soreness and damage reported in previous studies [4,7,9,22,25], BCAA supplementation alone was not sufficient to effectively inhibit muscle soreness and damage in the present study. This discrepancy might be due to differences in the exercise protocol (intensity and type) and the supplemental regimen (duration and dose). In a previous study by Shimomura *et al*., the authors recognized that the intensity was low in a squatting exercise where subjects used only their body weight because the changes in the levels of serum muscle damage markers, including CK and myoglobin, were very small over the three days following exercise [7,8]. On the other hand, repeated arm extensions with weight loads of 90% MVC in the present study caused a significant increase in serum muscle damage markers in the placebo group, thereby implying higher exercise intensity. The present findings with this higher intensity suggest that a combination of BCAA and taurine taken during high-intensity exercise may prevent severe muscle soreness and damage that cannot be attenuated by BCAA alone.

In addition to exercise intensity, the amount of oral BCAA intake is one of the important factors for preventing exercise-induced muscle soreness and damage. Shimomura *et al.* suggested that the BCAA dose should be adjusted according to body mass to at least 92–100 mg/kg because the inhibitive effects of BCAA on DOMS and muscle damage were greater in females than in males [7,8]. The BCAA dose in the present study should be sufficient because daily BCAA supplementation at 9.6 g/day worked out to 145.67 ± 5.3 mg/kg. Furthermore, the overall BCAA intake was probably sufficient because amino acid supplementation was from two weeks before to three days after exercise throughout the whole experimental period. Therefore, both the amount per body mass and the duration of BCAA supplementation in the present study might be sufficient for attenuating DOMS and muscle damage.

However, plasma BCAA concentrations were not altered by the BCAA supplementation in the present study. The two-week duration of BCAA supplementation prior to exercise was used to match the duration of taurine supplementation because this study was a double-blind trial. Indeed, a previous study conducted with college swimmers found no differences in plasma BCAA concentration after supplementation with 12 g/day BCAA for two weeks [27]. Hamada *et al.* reported that the plasma BCAA concentration in healthy humans significantly and rapidly increased and peaked at 30 min after a single BCAA dose; however, the plasma concentration returned to the basal level within 1–2 h [28] because of transport to the skeletal muscle [24]. Since blood sampling in the present study was done before each BCAA supplementation, the plasma BCAA concentration should have already returned to the basal level by the sampling time.

Taurine content in the skeletal muscle is also thought to be important for preventing muscle damage; however, neither the optimal duration nor the total dose of taurine has been clarified. We previously confirmed in rats that two weeks of oral taurine administration significantly increases taurine concentration in both the skeletal muscle and plasma in a dose-dependent manner [20,26]. In the present study, oral taurine administration at 6.0 g/day for two weeks significantly increased the plasma taurine concentration. Therefore, we suggest that the taurine concentration in the skeletal muscle in the present study might have been increased in line with the plasma level. However, a previous study with humans reported that seven days of oral taurine supplementation (5.0 g/day) did not change the taurine concentration in the skeletal muscle or in the plasma [21]. This discrepancy between the present results and those of previous studies with humans might be due to differences in the supplemental protocol. Therefore, an effective protocol for taurine supplementation, including dose and duration, to increase muscle taurine concentration as well as plasma level should be clarified in the future. Interestingly, Galloway *et al.* demonstrated that BCAA concentration in the skeletal muscle after exercise was significantly increased by oral taurine administration for seven days [21]. Although the mechanism to increase the muscular BCAA pool is unclear, it is one of the possible reasons why taurine might enhance the inhibitive effect of BCAA on muscle damage induced by ECC.

Oxidative stress-induced muscle damage has been shown to be associated with muscle soreness, and exercise-induced free radicals cause oxidative damage to cellular DNA. Radák *et al.* confirmed that the levels of 8-OHdG, a product of DNA oxidation, in the biopsied quadriceps femoris muscle of humans were significantly increased after 24 h of ECC when DOMS was present, suggesting that DNA damage occurred at the time of developing muscle soreness [29]. In the present study, significantly increased serum 8-OHdG levels were observed in the PLCB, BA, and TAU groups on Day 2 when DOMS peaked. The increased levels of plasma 8-OHdG were significantly decreased by the combined supplementation and tended to be lower than those achieved by taurine supplementation alone. Since we also observed in our previous study that taurine treatment significantly inhibited hepatic 8-OHdG levels in response to drug-induced oxidative stress [17], taurine might play a protective role in anti-DNA oxidation associated with DOMS in the skeletal muscle. To our knowledge, there is no evidence that BCAAs can suppress exercise-induced DNA damage in the skeletal muscle. However, patients with liver cirrhosis showed that chronic oral BCAA therapy significantly decreased urinary 8-OHdG excretion, suggesting that BCAAs could reduce oxidative stress-induced DNA damage in the skeletal muscle [30]. This might be a possible reason for the combined effect of BCAA and taurine on DOMS and muscle damage through protecting against DNA damage.

In addition to oxidative stress, intramuscular inflammation has also been considered a possible cause of DOMS [31]. To attenuate DOMS, it is important to inhibit the acute inflammatory response triggered by pro-inflammatory cytokines released from inflammatory cells following exercise [32]. Indeed, polymorphonuclear leukocytes are activated after ECC-induced DOMS and muscle damage [33]. Within several hours after exercise, circulating neutrophils rapidly invade damaged muscle. Thereafter, neutrophils within the damaged muscle are replaced by macrophages over the next 24 h and these macrophages produce pro-inflammatory cytokines [4,6]. A previous study reported that BCAA decrease the levels of Th1-derived cytokines (interferon-γ and interleukin-2) after high-intensity exercise, including triathlon and long-distance running [22]. Furthermore, taurine is an important factor in the neutrophil-related inflammatory response because it scavenges hypochlorous acid excreted from activated neutrophils and forms the less toxic taurine-chloramine [16,17]. Consequently, the production of pro-inflammatory mediators, such as prostaglandin E2 (PGE2), nitric oxide, and cytokines, from macrophages and lymphocytes are suppressed [34]. In particular, PGE2 has been considered a critical inflammatory mediator because it is produced by macrophages, sensitizes muscle afferent nociceptors [35], and is associated with the production of bradykinin, a substrate known to mediate muscle pain [36]. Although taurine-chloramine and PGE2 were not measured in the present study, we speculate that taurine *per se* may suppress PGE2 production in the arachidonate cascade via phospholipase A2 and cyclooxygenase 2 [37], as both of these enzymes are activated by increased [Ca^2+^]*i* levels and oxidative stress [38]. Thus, taurine might synergistically enhance the beneficial effects of BCAA for reducing DOMS and muscle damage via an anti-inflammatory/immune response. However, this hypothesis requires verification.

In terms of the “no pain, no gain” theory, the requirement of exercise-induced muscle soreness and an inflammatory response for muscle hypertrophy remains controversial. In the present study, the combination of BCAA and taurine suppressed DOMS and the levels of serum marker of oxidative stress. The general consensus is that muscle hypertrophy is induced during the recovery from damages to the microstructure of the muscle fiber and extracellular matrix [39]. Because exercise-induced symptoms including the production of inflammatory cytokine (interleukin-6; IL-6, and fibroblast growth factor-2), oxidative stress and DOMS usually occur during recovery, these responses have been suggested to be necessary for exercise-induced muscle hypertrophy [40,41]. Therefore, even if DOMS and muscle damage were effectively attenuated by the combination of BCAA and taurine supplementation, there is a possibility that muscle hypertrophy can be also be suppressed, and previous reports have shown that supplementations of taurine or multi-nutrient including BCAA and taurine could attenuate the productions of reactive oxygen species [16] and IL-6 [19]. On the other hand, Flann *et al.* evaluated whether exercise-induced symptoms including muscle soreness and damage are necessary events for muscle remodeling in humans [42]. They showed that the volume and strength of the quadriceps muscle and the muscular mRNA expression of the myogenic insulin-like growth factor-IEa that contributes to muscle regeneration were caused independently of muscle soreness and increase serum CK levels. Thus, DOMS and inflammation are not always necessary for muscle hypertrophy to occur. Furthermore, if exercise-induced DOMS and inflammation are efficiently attenuated, subjects can avoid unnecessary pain.

## Conclusion

This study confirmed that a combination of 3.2 g BCAA and 2.0 g taurine, three times a day, two weeks prior to and three days after exercise attenuates some subjective and objective markers of DOMS and muscle damage induced by high-intensity ECC, which could not have been influenced by BCAA or taurine supplementation alone. Therefore, combined supplementation with BCAA and taurine may be a useful strategy for attenuating DOMS and muscle damage and can help motivate beginners to continue an exercise program while assisting competitive athletes to train at higher intensity.

## Abbreviations

AEx: Immediately after exercise; ANOVA: Analysis of variance; AUC: Area under the curve; BA: Branched-chain amino acid and placebo 2 supplementation group; BCAA: Branched-chain amino acid; BEx: Before exercise; CIR: Upper arm circumference; CK: Creatine kinase; COMB: Branched-chain amino acid and taurine supplementation; Day1–4: 1–4 days after the exercise; DOMS: Delayed onset muscle soreness; ECC: Eccentric exercise; ICC: Intraclass correlation coefficient; IL-6: Interleukin-6; LDH: Lactate dehydrogenase; MVC: Maximal voluntary strength of isometric contraction; PGE2: Prostaglandin E2; PLCB: double-placebo control supplementation group; PRE: Prior to amino acid supplementation; TAU: Taurine and placebo 1 supplementation; VAS: Visual analogue scale; 8-OHdG: 8-hydroxydeoxyguanosine.

## Competing interests

The authors declare that they have no competing interests.

## Authors’ contributions

Significant manuscript writer: SGR, TM, and HO. Concept and design: SGR, TM, SM, YM, and HO. Data acquisition: SGR, TM, KI, HN, and SK. Data analysis and interpretation: SGR, TM, KI, HN, SK, YN, and HO. Statistical expertise: YN. Significant manuscript reviewer/reviser: SM, YM, and HO. All authors read and approved the final manuscript.
